# Homocysteine levels correlate with AVSS-RigiScan test parameters in men with erectile dysfunction

**DOI:** 10.1186/s12610-022-00181-9

**Published:** 2023-03-23

**Authors:** Xin Qian, Xing Tao, Yangyang Gong, Can Ran, Yougang Feng, Hongjian Liu

**Affiliations:** 1 Department of Urology, Suining Central Hospital, Suining, 629000 Sichuan Province China; 2 Faculty of Medicine, Zunyi Medical University, Zunyi, 563000 China

**Keywords:** Audio-visual sexual stimulation (AVSS) test, Erectile function (ED), RigiScan plus, Homocysteine, Stimulation sexuelle audiovisuelle (AVSS), Fonction érectile, RigiScan Plus, Homocystéine

## Abstract

**Background:**

Although elevated homocysteine levels have been shown to affect penile erection, the relationship between homocysteine and erection at the tip or base of the penis has not been extensively studied.

**Results:**

We found that homocysteine levels were negatively correlated with the average event rigidity of the base (*r* = -0.2225, *p* = 0.0142). Homocysteine levels were also negatively correlated with the average maximum rigidity of the base (*r* = -0.2164, *p* = 0.0171). In particular, homocysteine levels were negatively correlated with ∆ Tumescence of the tip (*r* = -0.1866, *p* = 0.0404). Similarly, homocysteine was negatively correlated with ∆ Tumescence of the base (*r* = -0.2257, *p* = 0.0128).

**Conclusion:**

Our data showed that homocysteine inhibits penile erection. At the same time, homocysteine levels were negatively correlated with the parameters of the AVSS-RigiScan test.

## Résumé

**Contexte:**

Bien qu’il ait été démontré que des niveaux élevés d’homocystéine affectaient l’érection pénienne, la relation entre homocystéine et érection à l’extrémité ou à la base du pénis n’a pas été étudiée de manière approfondie.

**Résultats:**

Nous avons constaté que les niveaux d’homocystéine étaient négativement corrélés avec la rigidité moyenne de la base (*r* = -0,2225, *p* = 0,0142). Les taux d’homocystéine étaient également négativement corrélés avec la rigidité maximale moyenne de la base (*r* = -0,2164, *p* = 0,0171). En particulier, les taux d’homocystéine étaient négativement corrélés avec la tumescence Δ de l’extrémité (*r* = -0,1866, *p* = 0,0404). De même, l’homocystéine était négativement corrélée avec la tumescence Δ de la base (*r* = -0,2257, *p* = 0,0128).

**Conclusions:**

Nos données ont montré que l’homocystéine inhibe l’érection pénienne. Dans le même temps, les niveaux d’homocystéine étaient négativement corrélés avec les paramètres du test AVSS-RigiScan.

## Introduction

Erectile dysfunction (ED), one of the most common types of male sexual dysfunction, is the consistent inability of the penis to achieve or maintain an erection sufficient for satisfactory sexual intercourse and is a chronic condition that affects men's physical and mental health [1]. According to the Massachusetts Male Aging Study (MMAS), ED affects half of men aged 40–70 years and up to 70% of older men [2]. The largest European multicentre population-based study of older men (40–79 years) reported that the prevalence of erectile dysfunction ranged from 6 to 64%, depending on the age group, and that the prevalence increased annually with age, with an average prevalence of 30% [3]. ED also has a high prevalence in China, and a study based on a five-item International Index of Erectile Function questionnaire showed that the prevalence of ED was 40.56% at an age of at least 40 years in 5210 men from 30 provinces and autonomies [4]. There are many risk factors for ED, including age, physical diseases, medications, lifestyles, and living conditions [5]. According to the available studies, ED is usually associated with systematic diseases such as diabetes mellitus, hyperlipidaemia, coronary artery disease, peripheral vascular disease, and cerebrovascular disease [6]. Atherosclerosis is a systemic pathological change of the blood vessels that also affects the cavernous arteries and may lead to altered blood flow at the penile level. Among the cardiovascular risk factors affecting the development of atherosclerosis, hyperhomocysteinemia (HHcys) plays a core role and is associated with oxidative stress and endothelial dysfunction [7].

RigiScan, a monitor of penile rigidity, has 2 testing modes: nocturnal and proactive. The nocturnal mode is mainly used for continuous monitoring during the nighttime sleep state, and the proactive mode is mainly used for monitoring by audio-visual sexual stimulation (AVSS) during the waking state. The NRT-RigiScan test and AVSS-RigiScan test have their own advantages in clinical application, and the correlation between the two tests is high [8]. The current majority view is that the AVSS-RigiScan test is simple, practical, quick and inexpensive and is suitable for initial aetiologic screening of ED patients in general. There is no standardized normative reference standard for the AVSS-RigiScan test, and most clinical centres refer to the NPTR-RigiScan diagnostic criteria. Wang et al. studied 1169 ED patients aged 18–67 years by the AVSS-RigiScan test and found that a basal rigidity of over 60% was sustained for at least 8.75 min, with an average event rigidity of the tip reaching at least 43.5% and that of the base reaching at least 50.5%; an average maximum rigidity of the tip reaching at least 62.5% and that of the base reaching at least 67.5%; ∆tumescence (increase in tumescence or maximum − minimum tumescence) of the tip measuring at least 1.75 cm and that of the base measuring at least 1.95 cm; and a total tumescence time measuring at least 29.75 min [9]. The number of times attaining total tumescence at least once can be used as a reference criterion for normal erectile hardness by AVSS, and AVSS testing after oral PDE5i is more objective and accurate in identifying psychological and organic ED [9].

Studies have shown that patients with high levels of Hcys are at increased risk of developing ED [10]. HHcys leads to reduced expression and activation of endothelial nitric oxide enzyme (NOS). A study by Giovannone concluded that the Hcys level is an early predictor of ED that is superior to Doppler ultrasound [11]; that study revealed that increased Hcys levels in patients with mild ED were present before abnormal Doppler ultrasound values were observed. Al-Hunayan et al. compared 97 patients exhibiting type 2 diabetes mellitus with vascular ED and 97 type 2 diabetic patients without ED in a case‒control study, demonstrating that HHcys is a major determinant of ED in diabetic patients [12]. A cross-sectional study by Salvio et al. who collected clinical data, Hcys levels, and penile ultrasound Doppler data from 126 patients with ED of arterial origin for analysis showed that Hcys levels were associated with penile blood flow velocity parameters in basal penile duplex ultrasound, demonstrating the role of Hcys levels in vasculogenic ED [13].

Therefore, the present study aimed to investigate the correlation between serum homocysteine levels and audio-visual sexual stimulation and RigiScan™ (AVSS-RigiScan) test parameters in men with erectile dysfunction to further clarify the relationship between serum homocysteine levels and erectile dysfunction in men and to provide reliable data support for clinical application.

## Materials and methods

### Inclusion and exclusion criteria

Ninety-nine patients with a diagnosis of erectile dysfunction who attended the male outpatient clinic of Suining Central Hospital from October 2021 to March 2022 were selected for inclusion in the case group. Twenty-two men without sexual dysfunction who underwent a health examination or premarital examination during the same period were included in the control group. Participants were grouped and analysed retrospectively. All participants provided informed consent (which included a statement of confidentiality of responses and the right to stop answering at any time) and were given answers to any questions regarding the significance of the survey items. The inclusion criteria for participants were as follows: age from 40 to 75 years; regular sexual activity within the last six months; and International Index of Erectile Function (IIEF-5) ≤ 21. Subjects were excluded from the study according to the following criteria: abnormal sex hormones, cardiovascular accident within the last 6 months, medication affecting serum homocysteine levels or testosterone levels within the last 3 months, history of genital surgery, and history of genital trauma.

### Study design

A detailed history, physical examination, laboratory evaluation, and erectile function examination (i.e., audio-visual sexual stimulation (AVSS) test) was administered to all participants in the study. All patients' general information included age, IIEF-5 score, and Erection Hardness Scale (EHS) score. The following biochemical parameters were considered: glucose, total cholesterol (TC), triglycerides (TGs), high-density lipoprotein (HDL) cholesterol, low-density lipoprotein (LDL) cholesterol, apolipoprotein A1 (ApoA1), apolipoprotein B (ApoB), lipoprotein (a), fructosamine (FMN), homocysteine (Hcys), and testosterone (T). Normal ranges for adults were 3.88–6.38 mmol/L (glucose), < 5.17 mmol/l (cholesterol), < 1.70 mmol/L(TG), > 1.04 mmol/L (HDL), < 3.12 mmol/L (LDL), 1.00–1.60 g/L (ApoA1), 0.6–1.1 g/L (ApoB), ≤ 300 mg/l (lipoprotein(a)), 1.10–2.15 mmol/L (FMN), < 15 µmol/l(Hcys), 4.94–32.01 nmol/l (T).

The audio-visual sexual stimulation (AVSS) test was monitored by the RigiScan™ plus device using the RigiScan™ plus proactive mode. Participants were monitored in a quiet, comfortable, warm, softly lit room without external disturbances, with a calm and comfortable mood, free of all distractions. The patient was given 20 mg of oral vardenafil, and after 30 min, the RigiScan™ plus was fixed on the patient's right thigh according to the instructions. The 3D VR glasses were put on and adjusted, and the basal values were recorded for 15 min with quiet rest. A 30-min erotic video was shown to each patient individually, followed by stimulation of rigidity and tumescence for the next 30 min. All recorded data were transferred to a computer and analysed using RigiScan software.

### Statistical analysis

The Statistical Package for Social Sciences (SPSS) version 18.0 (SPSS Inc., Chicago, IL) for Microsoft Windows was used for the statistical analysis of the data. Data baselines are expressed as the mean ± standard deviation (SD). The Mann‒Whitney U test was used to compare the case group and control group between groups. The data were divided into subgroups 1 (Hcys ≥ 15 µmol/l) and 2 (Hcys < 15 µmol/l) according to homocysteine levels. The Mann‒Whitney U test was performed for comparisons between subgroups. Pearson's test or Spearman's test (normal or nonnormal distribution) was used to test the correlation between two variables. The threshold for significance was *p value* < 0.05.

## Results

All participants underwent general data collection and biochemical parameter assessments and completed the AVSS-RigiScan test. In the baseline analysis, the ED and non-ED groups were divided according to IIEF-5. The clinical characteristics of participants with ED and participants with normal erectile function are shown in Table 1. The mean age of the ED patients was 49.38 ± 0.66 years, and the mean homocysteine value was 14.03 ± 0.50 µmol/l. The mean age of the 22 healthy individuals in the control group was 37.14 ± 1.86 years, and the mean homocysteine value was 11.70 ± 0.53 µmol/l. The non-ED group had higher homocysteine levels than the ED group, with a statistically significant difference between the two groups (*p* = 0.0098). The data were divided into subgroup 1 (Hcys ≥ 15 µmol/l) and subgroup 2 (Hcys < 15 µmol/l) according to homocysteine levels. Table 2 shows a comparison of the parameters of the AVSS-RigiScan test between the two groups. The results for average event rigidity of the tip, average maximum rigidity of the tip, ∆ tumescence of the tip, and ∆ tumescence of the base were all lower for subgroup 1 than for subgroup 2 but were not significantly different. The average event rigidity of the base was lower in subgroup 1 than in subgroup 2, with a statistically significant difference (*p* = 0.0034). The average maximum rigidity of the base was lower in subgroup 1 than in subgroup 2, and the difference was statistically significant (*p* = 0.0007).

**Table 1:** Comparison of clinical and laboratory data between the non-ED group and the ED group

	**Non-ED group (*N* = 22)**	**ED group (*N* = 99)**	**Mann‒Whitney U**	*p*
Age (years)	37.14 ± 1.86	49.38 ± 0.62	326.5	< 0.0001
IIEF-5	22.91 ± 0.23	11.83 ± 0.49	0	< 0.0001
EHS	2.82 ± 0.11	2.12 ± 0.07	505.5	< 0.0001
Glucose (mmol/L)	5.59 ± 0.18	6.53 ± 0.23	708.5	0.0107
TC (mmol/L)	5.38 ± 0.23	5.63 ± 0.11	930	0.2868
TG (mmol/L)	2.38 ± 0.57	2.03 ± 0.12	1028	0.6843
HDL (mmol/L)	1.08 ± 0.04	1.18 ± 0.029	865.5	0.1339
LDL (mmol/L)	3.25 ± 0.17	3.44 ± 0.09	953.5	0.3643
ApoA1 (g/L)	1.49 ± 0.04	1.51 ± 0.03	1066	0.8798
ApoB (g/L)	0.88 ± 0.04	0.96 ± 0.02	878.5	0.1581
lipoprotein(a) (mg/L)	137.1 ± 20.58	193.7 ± 18.99	997	0.5386
FMN (mmol/L)	1.84 ± 0.04	1.96 ± 0.03	760	0.0273
T (mmol/L)	21.50 ± 1.48	18.77 ± 0.74	826	0.0777
Hcys (µmol/L)	11.70 ± 0.53	14.03 ± 0.50	704	0.0098

Data are expressed as the mean ± standard deviation; *P* values for the ED group and non-ED group were derived from Mann‒Whitney U test (for continuous dependent variables)

*ED* Erectile function, *IIEF-5* International Index of Erectile Function, *EHS* Erection Hardness Scale, *TC* Total cholesterol, *TG* Triglycerides, *HLD* High-density lipoprotein cholesterol, *LDL* Low-density lipoprotein cholesterol, *ApoA1* apolipoprotein A1, *ApoB* Apolipoprotein B, *FMN* Fructosamine, *T* Testosterone, *Hcys* Homocysteine

**Table 2:** Instrumental data (AVSS-RigiScan) of patients with normal versus high levels of homocysteine

	**Subgroup 1 (hcy ≥ 15 µmol/L)**	**Subgroup 2 (hcy < 15 µmol/L)**	**Mann‒Whitney U**	*p*
average event rigidity of the tip (%)	30.80 ± 2.70	35.33 ± 1.67	1083	0.0904
average event rigidity of the base (%)	39.87 ± 1.92	48.90 ± 1.68	876.5	0.0034
average maximum rigidity of the tip (%)	58.23 ± 2.63	64.15 ± 1.86	1061	0.0679
average maximum rigidity of the base (%)	63.07 ± 2.38	73.49 ± 1.58	797.5	0.0007
∆Tumescence of the tip (cm)	1.93 ± 0.15	2.27 ± 0.10	1051	0.0593
∆Tumescence of the base (cm)	2.32 ± 0.11	2.58 ± 0.08	1053	0.0611

Data are expressed as the mean ± standard deviation; *P* values for Subgroup 1 and Subgroup 2 were derived from the Mann‒Whitney U test

*AVSS* Audio-visual sexual stimulation, *hcy* Homocysteine, *∆Tumescence* Increase in tumescence or maximum − minimum tumescence

**Fig. 1:**
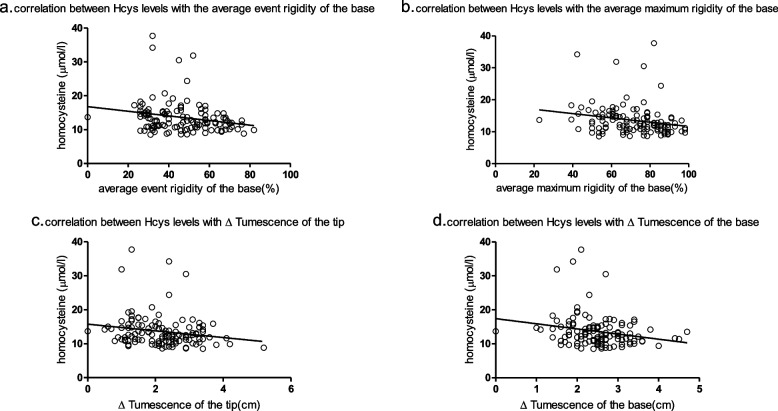
Correlation between Hcys levels and AVSS-RigiScan test parameters. **a** Hcys levels were negatively correlated with the average event rigidity of the base (*r* = -0.2225; *p* = 0.0142). Hcys: Homocysteine. **b** Hcys levels were negatively correlated with the average maximum rigidity of the base (*r* = -0.2164; *p* = 0.0171). Hcys: Homocysteine. **c** Hcys levels were negatively correlated with ∆Tumescence of the tip (*r* = -0.1866, *p* = 0.0404). Hcys: homocysteine. ∆Tumescence: increase in tumescence or maximum − minimum tumescence. **d** Hcys levels were negatively correlated with ∆ Tumescence of the base (*r* = -0.2257, *p* = 0.0128). Hcys: Homocysteine. ∆Tumescence: increase in tumescence or maximum − minimum tumescence

Table 3 shows the correlation analysis between homocysteine and the basic clinical information and biochemical parameters. It can be concluded that homocysteine was positively correlated with age (*r* = 0.2366, *p* = 0.009) and negatively correlated with the IIEF-5 score (*r* = -0.2123, *p* = 0.0194). Table 4 shows the relationship between homocysteine values and AVSS-RigiScan test results. It can be seen that the current data do not allow for a linear correlation between homocysteine and the average event rigidity and average maximum rigidity of the tip. We found that homocysteine levels were negatively correlated with the average event rigidity of the base (*r* = -0.2225, *p* = 0.0142) (Fig. 1a). Homocysteine levels were negatively correlated with the average maximum rigidity of the base (*r* = -0.2164, *p* = 0.0171) (Fig. 1b). In particular, homocysteine levels were negatively correlated with ∆Tumescence of the tip (*r* = -0.1866, *p* = 0.0404) (Fig. 1c). Similarly, homocysteine was negatively correlated with ∆ Tumescence of the base (*r* = -0.2257, *p* = 0.0128) (Fig. 1d). From Table 5, we can conclude that the average event rigidity of the base was negatively correlated with age (*r* = -0.2088, *p* = 0.0215), glucose values (*r* = -0.202, *p* = 0.0263), and homocysteine levels (*r* = -0.2225, *p* = 0.0142). With the current data, we cannot draw a linear correlation between average event rigidity of the base and cholesterol, triglycerides, HDL, LDL, lipoprotein A1, lipoprotein B, lipoprotein (a), fructosamine, and testosterone at this time.

**Table 3:** Correlation coefficients of HCY values with clinical parameters

	**r**	**95% CI**	*p*
Age (years)	0.237	0.061–0.398	0.009
IIEF-5	-0.212	-0.377- -0.035	0.020

*HCY* Homocysteine, *IIEF-5* International Index of Erectile Function

**Table 4:** Correlation coefficients of HCY values with AVSS-RigiScan test parameters

	**r**	**95% CI**	*p*
average event rigidity of the tip (%)	-0.158	-0.327–0.021	0.084
average event rigidity of the base (%)	-0.223	-0.386—-0.046	0.014
average maximum rigidity of the tip	-0.165	-0.334–0.014	0.071
average maximum rigidity of the base	-0.216	-0.380—-0.040	0.017
∆ Tumescence of the tip	-0.187	-0.353—-0.008	0.040
∆ Tumescence of the base	-0.226	-0.389—-0.049	0.013

*HCY* Homocysteine, *AVSS* Audio-visual sexual stimulation, *∆Tumescence* Increase in tumescence or maximum − minimum tumescence

**Table 5:** Correlation coefficients of the average event rigidity of the base by clinical parameter

	**r**	**95% CI**	*p*
Age	-0.209	-0.373—-0.031	0.022
glucose	-0.202	-0.367—-0.024	0.026
TC	0.095	-0.085–0.269	0.299
TG	0.021	-0.159–0.198	0.823
HDL	-0.075	-0.251–0.105	0.412
LDL	0.115	-0.065–0.288	0.208
ApoA1	-0.109	-0.282–0.071	0.233
ApoB	0.095	-0.085–0.269	0.301
lipoprotein(a)	-0.028	-0.205–0.152	0.764
FMN	-0.047	-0.223–0.133	0.612
T	-0.029	-0.206–0.150	0.752
Hcys	-0.2225	-0.3857 to -0.04579	0.0142

*TC* Total cholesterol, *TG* Triglycerides, *HLD* High-density lipoprotein cholesterol, *LDL* low-density lipoprotein cholesterol, *ApoA1* Apolipoprotein A1, *ApoB* Apolipoprotein B, *FMN* Fructosamine, *T* Testosterone, *Hcys* Homocysteine

## Discussion

The relationship between homocysteine and erectile dysfunction was discussed as early as 2004 when Jones R. W et al. established a rabbit model of erectile dysfunction due to HHcys [14]. The results of several previous studies have shown a significant correlation between HHcys and erectile dysfunction. The association between homocysteine, vitamins, and folic acid and erectile dysfunction was evaluated in a cross-sectional study based on 1318 participants by Chen et al., which showed a significant association between homocysteine and erectile dysfunction, most significantly in men over 60 years old and in those living alone (single) [7, 15]. A recent study by Wang et al. based on 119 patients with erectile dysfunction showed that patients with erectile dysfunction with HHcys were 13.42 times more likely to develop vasogenic erectile dysfunction than patients without HHcys [16]. Giovannone et al. included 431 participants in their study and showed that plasma homocysteine levels were associated with the severity of erectile dysfunction and that the homocysteine level was expected to be a predictor of the development of erectile dysfunction [11]. The results of this study were consistent with previously reported findings, with a statistically significant difference in the erectile dysfunction group compared to the control group (*p* = 0.0098).

Since the invention of the RigiScan by Bradley et al. in 1985 [17], nocturnal penile erectile rigidity tests and audio-visual sexual stimulation tests have been widely used in the field of urology and andrology. The RigiScan, which is produced by GOTOP in the United States, is used to assess the aetiology and severity of ED and can objectively and effectively identify psychological and organic ED and has been included in the official ED treatment guidelines of the American and European Association of Urology [18]. The use of RigiScan™ in the diagnosis of nocturnal penile erection and rigidity has been recognized as an effective tool for distinguishing psychological erectile dysfunction from organic erectile dysfunction [9]. In the previously reported literature on the association between homocysteine and erectile dysfunction, the diagnostic application of RigiScan™ in nocturnal penile tumescence and rigidity (NPTR), was used in most studies to evaluate erectile function, and few studies used AVSS-RigiScan for erectile function evaluation. There are no AVSS-RigiScan evaluation criteria in any of the guidelines, including the EAU. Therefore, few researchers have used AVSS-RigiScan to evaluate erectile function in literature reports. In the diagnosis of ED, AVSS-RigiScan is a more widely used tool than NPTR-RigiScan. Compared to NPTR testing, penile erection during AVSS testing is more similar to erotic and reflex erection activity, which is relatively simple, economical, less time-consuming, more physiologically consistent, and unaffected by sleep [8, 19–21]. In a study by Wang et al., standardized Chinese AVSS-RigiScan evaluation criteria were established by the AVSS-RigiScan test used in combination with oral phosphodiesterase-5 inhibitors [9]. It has been shown that administration of phosphodiesterase-5 inhibitors before AVSS-RigiScan testing not only avoids or reduces the shortcomings of the routine AVSS-RigiScan but also improves its diagnostic properties [20, 22, 23]. Therefore, in this study, the AVSS-RigiScan test was performed to evaluate erectile function, and the participants were given 20 mg of oral vardenafil 30 min before the test. In our study, the aim was to investigate the correlation between serum homocysteine levels and AVSS-RigiScan test parameters in men with erectile dysfunction. The results of the correlation analysis in Table 3 also show that homocysteine levels were negatively correlated with the average event rigidity of the base (*r* = -0.2225, *p* = 0.0142) and the average maximum rigidity of the base (*r* = -0.2164, *p* = 0.0171). Table 5 also shows that the average event rigidity of the base was negatively correlated with age (*r* = -0.2088, *p* = 0.0215), glucose (*r* = -0.202, *p* = 0.0263) and homocysteine (*r* = -0.2225, *p* = 0.0142).

According to the present study, oxidative stress is the main biochemical mechanism leading to homocysteine-induced cell damage and endothelial dysfunction. HHcys induces ED by altering virtually every component of NO metabolism, including NOS expression, localization, activation, and activity. HHcys significantly reduces the expression of endothelial nitric oxide synthase (eNOS) protein in a dose-dependent manner. Initially, endothelial cells can increase NO synthesis and release to protect themselves and detoxify HHcys, which in turn leads to the formation of the S-nitroso homocysteine, a potent vasodilator. However, this defence mechanism is limited, and prolonged exposure to HHcys eventually leads to impaired basal NO production, free radical formation, and subsequent endothelial damage [24]. Reduced NO production and decreased cGMP concentrations lead to decreased vascular smooth muscle diastolic function, as well as impaired endothelium-dependent vasodilatory responses, causing endothelial dysfunction, which leads to various vascular diseases, including ED [25].

## Limitations of the study

We should also be concerned about some limitations of this study. First, this study was a single-centre study, which may have implications for external validation. Second, the relatively small sample size may limit the generalizability of the results. Third, repeated AVSS-RigiScan tests were performed to ensure diagnostic accuracy. In addition, a comprehensive series of blinded validation studies are necessary to determine the relationship between homocysteine and AVSS-RigiScan test parameters. What we also need to acknowledge is the lack of psychometric tests to assess erectile function, which is a study limitation. For participants with abnormal AVSS-RigiScan test results, we did not perform further examinations, including the NPTR-RigiScan test and noninvasive arterial Doppler ultrasonography.

## Conclusion

We can conclude that homocysteine levels are inhibitory to penile erectile function and negatively correlate with average event rigidity of the base and average maximum rigidity of the base in the AVSS-RigiScan test. This also confirms that homocysteine is a potential indicator for the diagnosis of ED.

## Data Availability

The datasets used and/or analysed during the current study are available from the corresponding author on reasonable request.

## Abbreviations

AVSS: Audio-visual sexual stimulation
ED: Erectile function
MMAS: Massachusetts Male Aging Study
Hhcys: Hyperhomocysteinemia
Hcys: Homocysteine
SAM: S-adenosylmethionine
SAH: S-adenosylhomocysteine
SPSS: Statistical Package for Social Sciences
NPTR: Nocturnal penile tumescence and rigidity
IIEF-5: International Index of Erectile Function-5
EHS: Erection Hardness Scale
NOS: Nitric oxide enzyme
eNOS: Endothelial nitric oxide synthase
TC: Total cholesterol
TG: Triglycerides
HLD: High-density lipoprotein cholesterol
LDL: Low-density lipoprotein cholesterol
ApoA1: Apolipoprotein A1
ApoB: Apolipoprotein B
FMN: Fructosamine
T: Testosterone
